# Haploinsufficiency of Def Activates p53-Dependent TGFβ Signalling and Causes Scar Formation after Partial Hepatectomy

**DOI:** 10.1371/journal.pone.0096576

**Published:** 2014-05-06

**Authors:** Zhihui Zhu, Jun Chen, Jing-Wei Xiong, Jinrong Peng

**Affiliations:** 1 Key Laboratory for Molecular Animal Nutrition, Ministry of Education, College of Animal Sciences, Zhejiang University, Hangzhou, China; 2 College of Life Sciences, Zhejiang University, Hangzhou, China; 3 Institute of Molecular Medicine, Peking University, Beijing, China; National University of Singapore, Singapore

## Abstract

The metazoan liver exhibits a remarkable capacity to regenerate lost liver mass without leaving a scar following partial hepatectomy (PH). Whilst previous studies have identified components of several different signaling pathways that are essential for activation of hepatocyte proliferation during liver regeneration, the mechanisms that enable such regeneration to occur without accompanying scar formation remain poorly understood. Here we use the adult zebrafish liver, which can regenerate within two weeks following PH, as a new genetic model to address this important question. We focus on the role of Digestive-organ-expansion-factor (Def), a nucleolar protein which has recently been shown to complex with calpain3 (Capn3) to mediate p53 degradation specifically in the nucleolus, in liver regeneration. Firstly, we show that Def expression is up-regulated in the wild-type liver following amputation, and that the *def^hi429/+^* heteroozygous mutant (*def^+/−^*) suffers from haploinsufficiency of Def in the liver. We then show that the expression of pro-inflammatory cytokines is up-regulated in the *def^+/−^* liver, which leads to distortion of the migration and the clearance of leukocytes after PH. Transforming growth factor β (TGFβ) signalling is thus activated in the wound epidermis in *def^+/−^* due to a prolonged inflammatory response, which leads to fibrosis at the amputation site. Fibrotic scar formation in *def^+/−^* is blocked by the over-expression of Def, by the loss-of-function of p53, and by treatment with anti-inflammation drug dexamethasone or TGFβ-signalling inhibitor SB431542. We finally show that the Def- p53 pathway suppresses fibrotic scar formation, at least in part, through the regulation of the expression of the pro-inflammatory factor, high-mobility group box 1. We conclude that the novel Def- p53 nucleolar pathway functions specifically to prevent a scar formation at the amputation site in a normal amputated liver.

## Introduction

Liver regeneration refers to the process of regaining liver mass by compensatory growth after partial hepatectomy (PH) or toxic injury [1]–[3], and previous molecular and genetic studies have revealed the involvement of cytokine- and growth factor-mediated pathways in its regulation. Among these, interleukin (IL)-6 and hepatocyte growth factor (HGF) and their related signalling molecules are two well-studied pathways that have been shown to enable hepatocytes to overcome cell-cycle checkpoint controls [1]–[3]. Transcription factors, such as c-Jun, c-Fos, c-Myc, NF-κB, STAT3 and C/EBP, are also mobilised during liver regeneration [4]. It is noticed that the healing of the resection site in a normal healthy liver after PH is surprisingly not accompanied with fibrotic scar formation [5]. This contrasts to many other types of wound healing (e.g skin) which often leaves a fibrotic scar at the wounding site. The scarless wound healing serves, in fact, as the key basis for liver operation/transplantation. Scar formation is considered to be a consequence of the prolonged inflammation in the wound epidermis [5]. Interestingly, while the vast majority of previous studies have focused on the initiation, progression and termination of liver regeneration after PH, little work has been carried out on the mechanisms that underlie the scarless repair of the amputation site after PH [5].

In recent years, zebrafish has been used as a model to study the development and regeneration of the liver [6]–[8], and studies have shown that the amputated livers of wild-type zebrafish regained their mass within 15 days post PH [9], [10]. Digestion-organ-expansion-factor (Def) is a novel nucleolar factor that is conserved across species, from yeasts to humans [11]–[13]. In the zebrafish, the loss of Def function in the *def^hi429/hi429^* mutant conferred a phenotype characterised by a smaller liver, a shortened exocrine pancreas and thinner intestines [13]. Our recent studies showed that Def complexes with calpain 3 (Capn3) to mediate p53 degradation in the nucleolus in both human and zebrafish cells [14]. This finding defined a unique novel p53 degradation pathway, the Def-CAPN3-p53 pathway, in the nucleolus, and also explained why p53 protein specifically accumulates in the nucleolus in the *def^hi429/hi429^* homozygous mutant. Up-regulated p53 then selectively up-regulates the expression of p53 downstream genes, including *Δ113p53* and *p21* to cause cell-cycle arrest, which results in hypoplasia of the digestive organs in the mutant [13].

In this study, we examined the capacity of *def^+/−^* heterozygous fish to undergo liver regeneration after PH and found that Def haploinsufficiency in the *def^+/−^* strain activates a p53-dependent, inflammation-mediated transforming growth factor β (TGFβ) pathway that causes scar formation at the amputation site after PH in zebrafish. This function of the Def- p53 pathway is probably achieved partially through the regulation of the expression of high-mobility group box 1 (HMGB1), a pro-inflammatory pathway.

## Results

### The *def^+/−^* Zebrafish Is Defective in Recuperating the Lobe Structure At the Amputation Site after PH

**Figure 1:**
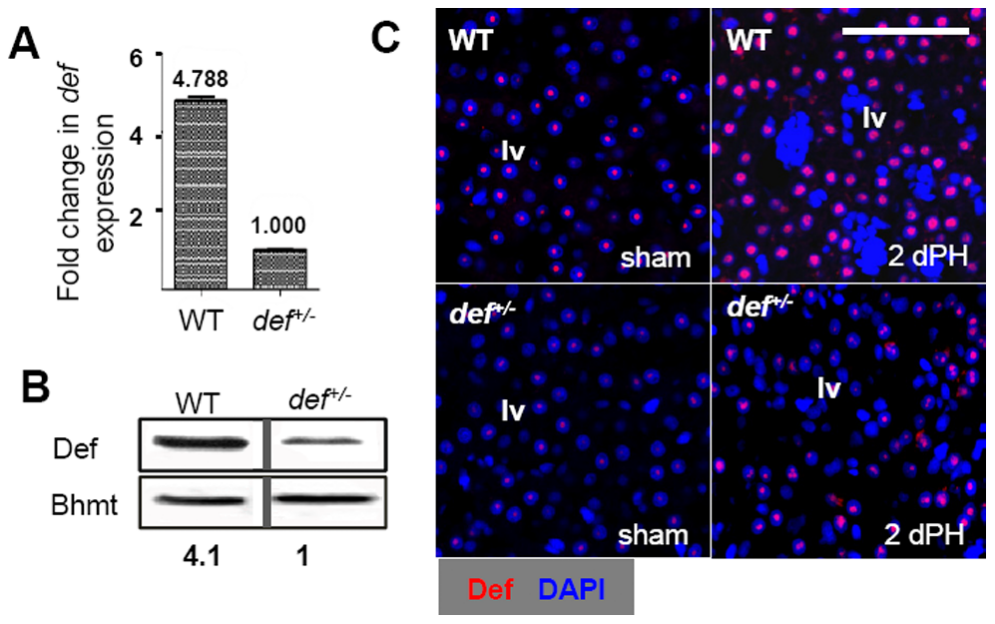
*def^+/−^* liver suffers from haploinsufficiency of Def. (*A*) qPCR analysis of *def* transcripts in adult wild-type and *def^+/−^* liver. The expression of *def* was normalised against *elf1a* and is shown as the fold change in expression, with the value from *def^+/−^* being set at 1. The values plotted represent the means ± standard errors of the mean. (*B*) Western blotting analysis of the Def protein. Bhmt: loading control. Densitometry was performed using Photoshop. The value of Def was obtained by normalising against Bhmt expression. The relative value of Def protein in *def^+/−^* was set at 1. (*C*) Representative images of immunostaining of Def (red) in wild-type and *def^+/−^* liver 2 days after PH. lv, liver tissue. Scale bar: 100 µm (*C*).

Our previous study showed that Def was expressed in the adult zebrafish liver [13]. Here, we compared the levels of *def* transcripts and Def protein, respectively, in the livers of adult wild-type and *def^+/−^* zebrafish and found that the levels of *def* transcripts were approximately 4.8-fold (Figure 1A) and those of Def protein approximately four fold (Figure 1B) lower in the *def^+/−^* mutant, demonstrating that the *def^+/−^* strain is a typical haploinsufficient mutant. Interestingly, we found that the level of Def was up-regulated in the nucleoli of the livers of both wild-type and *def^+/−^* adults 2 days after PH, but the staining intensity of Def was apparently much stronger in the wild-type (Figure 1C, Figure S1A). Despite of this, a *def^+/−^* fish grows to its adulthood normally without showing an overt phenotype (data not shown). Therefore, the *def^+/−^* fish allowed us to assess the effect of haploinsufficiency of Def on liver regeneration after PH.

**Figure 2:**
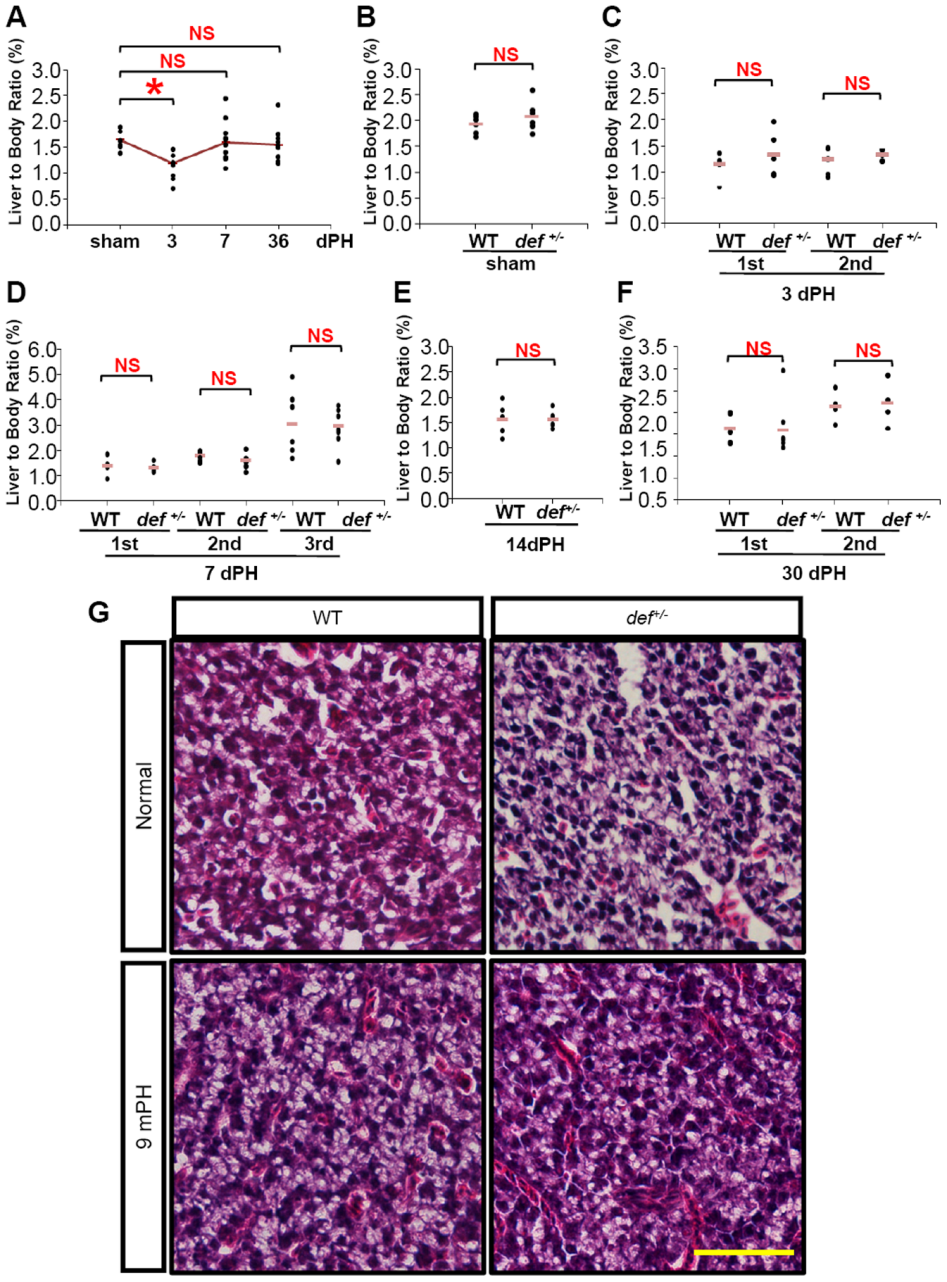
The liver-to-body ratio of the regenerated livers did not differ significantly between the wild-type and *def^+/−^* mutant fish after PH. (*A*) Liver-to-body ratio of the wild-type sham control and amputated wild-type liver at 3, 7 and 36 days after PH (n≥5). (*B*) Liver-to-body ratios of the sham controls (n≥5) for the wild-type and *def^+/−^* mutant fish. (*C–F*) Comparison of the liver-to-body ratios of the amputated wild-type and *def^+/−^* livers 3 days after PH (*C*), 7 days after PH (*D*), 14 days after PH (*E*) and 30 days after PH (*F*) as indicated (n≥5 in each case). NS: not significant; *: *p*<0.05. (*G*) Representative images of haematoxylin-eosin staining of sections across the wild-type or *def^+/−^* hepatic parenchyma without performing PH or 9 months after PH. Scale bar: 50 µm.

**Figure 3:**
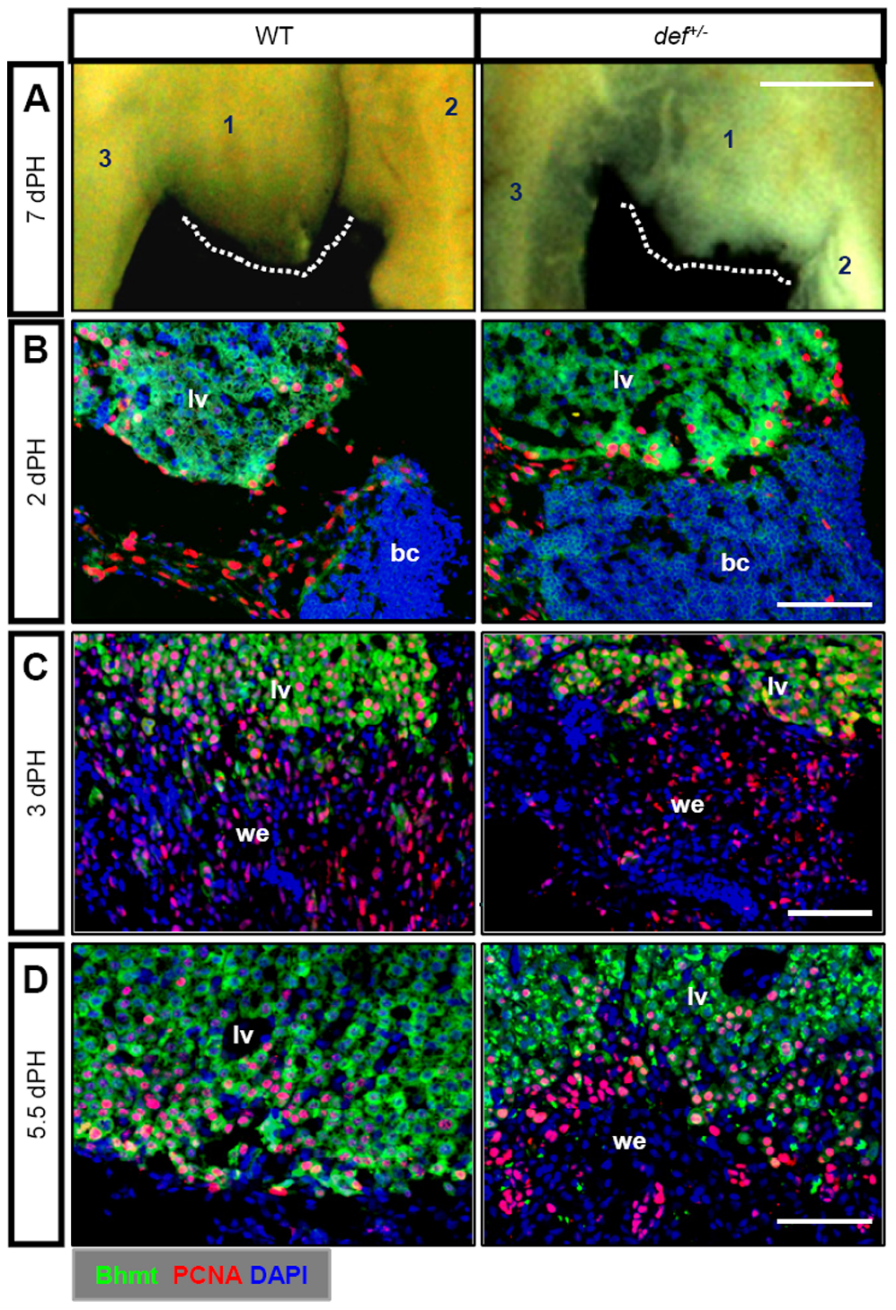
Def haploinsufficiency results in a failure in the remodelling of the wound epidermis to liver tissue. (*A*) Ventral view of the gross morphology of an amputated adult wild-type and *def^+/−^*liver 7 days after PH. The dotted white line indicates the amputation site. 1: ventral lobe, 2: left dorsal lobe, 3: right dorsal lobe. (*B–D*) Representative images of co-immunostaining of PCNA and Bhmt to compare the process of epithelialisation of the amputation site 2 days after PH (*B*), wound epidermis formation 3 days after PH (*C*), and wound epidermis remodelling 5.5 days after PH (*D*) between the wild-type and the *def^+/−^* mutant. Nuclei were stained with 4',6-diamidino-2-phenylindole (DAPI) (blue). In each case or at each time-point, more than 10 sections from at least three wild-type or *def^+/−^* mutant fish were examined. bc, blood clot; lv, liver tissue; we, wound epidermis. Scale bar: 5 mm (*A*) and 75 µm (*B–D*).

An adult zebrafish liver comprises two dorsal lobes and one ventral lobe [9], [15]. We surgically removed almost the entire ventral lobe from 5 to 7-month-old wild-type and *def^+/−^* adults and found that, similar to results reported for wild-type livers [7], [9], [10], *def^+/−^* fish regained their liver-to-body ratio at 7 days after PH (Figure 2A–F). In addition, haematoxylin andeosin (H&E) staining revealed no obvious structural changes in the liver parenchyma between wild-type and *def^+/−^* strains 9 months after PH (Figure 2G). We then compared the gross morphology of the liver at the amputation site 3, 7 and 21 days after PH (Figure S2). As expected, the majority of the resected ventral liver lobes (12 out of 15) in the wild-type fish recovered their lobe structure, characterised by a new smooth round lobe tip at the amputation site as early as 7 days after PH (Figure 3A; Figure S2), although the new lobe did not regain its original size up to 60 days after PH (data not shown). In contrast, ∼85% (11/13) of *def^+/−^* fish exhibited an uneven surface and lacked a lobe-like structure at the amputation site 7 days after PH (Figure 3A; Figure S2).

### The Wound Epidermis in *def^+/−^* Fish Failed To Be Remodelled and Finally Formed A Fibrotic Scar At the Amputation Site after PH

The previous result suggested that *def^+/−^* fish were unable to regenerate the liver structure at the amputation site after PH. The wound healing process can be divided into four sequential phases: haemostasis, inflammation, proliferation and remodelling/maturation [16]. To follow the healing process at the amputation site after PH, we simultaneously assessed cell-cycle entry (by following the nuclear incorporation of 5-ethynyl-2'-deoxyuridine (EdU), a marker for the S-phase) and cell apoptosis (using the terminal deoxynucleotidyl transferase dUTP nick end labeling (TUNEL) assay), together with immunostaining using betaine–homocysteine S-methyltransferase (BhmtHMT) (to mark hepatocytes) [17] antibody at different time points. At 12, 24 and 36 h after PH, we observed no obvious differences in terms of blood clot formation, cell apoptosis or proliferation between the wild-type and *def^+/−^*fish, except that the blood clot was almost detached from the amputation site in the wild-type but was still attached to the liver mass in the *def^+/−^* fish 36 h after PH (Figure S3).

We then performed immunostaining with the proliferating cell nuclear antigen (PCNA) in the background of the transgenic reporter fish line *Tg(fabp10a::dsRed)*, in which hepatocytes were genetically labelled by red fluorescent protein [18]. Proliferating cells were rarely observed either at the edge of or in the parenchyma in the sham control at 3 days after PH (Figure S4). In contrast, immunostaining with PCNA and Bhmt showed that the number of proliferating cells (PCNA-positive cells, PCNA^+^) was increased and that they were mainly detected near the amputation site in a similar pattern in both the wild-type and *def^+/−^* fish at 2 days after PH (Figure 3B). At 3 days after PH, in addition to active proliferating hepatocytes, a new cell mass that was Bhmt-negative (Bhmt^−^) appeared next to the amputation site in both the wild-type and *def^+/−^* fish, although fewer such cells were found in the *def^+/−^* fish (Figure 3C; Figure S1B). The last step of wound healing is the remodelling of the wounded site to restore the tissue structure [19]. Immunostaining with PCNA and Bhmt showed that, by 5.5 days after PH, almost all of the PCNA^+^ cells around the amputation site in the wild-type fish were also Bhmt^+^ (Figure 3D), indicating that the wound epidermis had been successfully remodelled to the liver tissue. In contrast, many PCNA^+^ cells in the *def^+/−^* livers were still Bhmt^−^ (Figure 3D).

**Figure 4:**
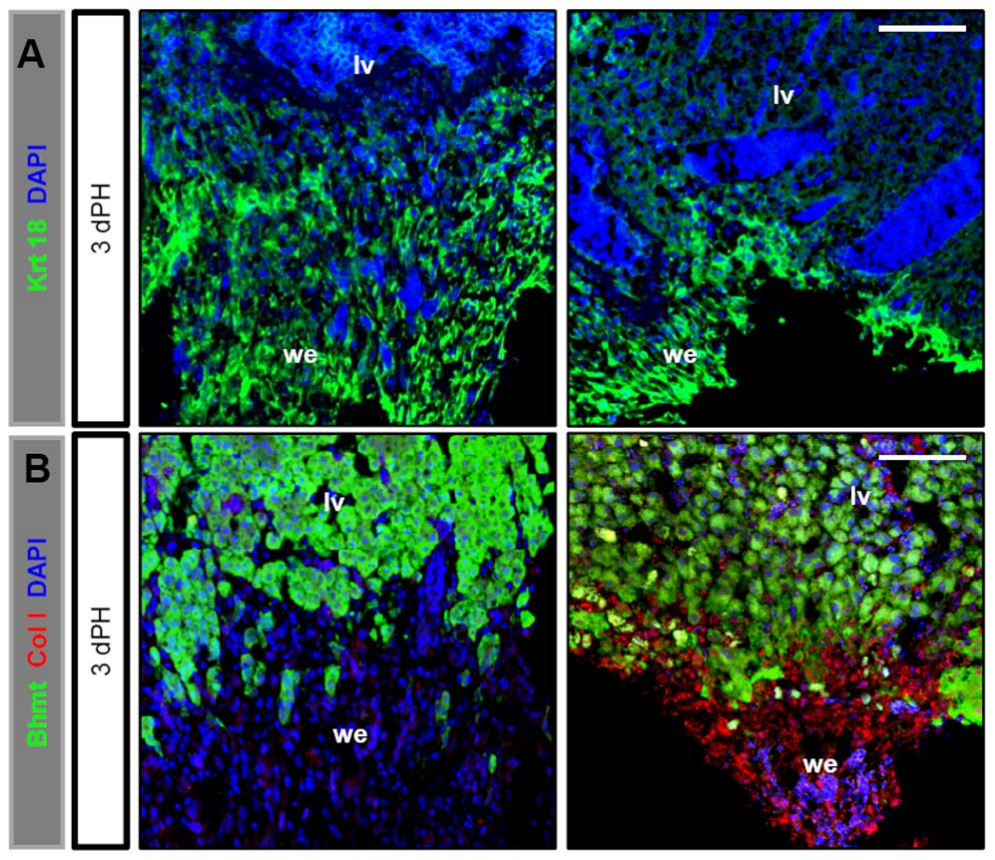
Def haploinsufficiency results in accumulation of ColI at the amputation site after PH. (*A,B*) Representative images of immunostaining of keratin 18 (green) (*A*) and co-immunostaining of ColI (red) and Bhmt (green) (*B*) at the amputation site in wild-type and *def^+/−^* strains 3 days after PH. Nuclei were stained with DAPI (blue). At each time-point, more than 10 sections from at least three wild-type or *def^+/−^* mutant fish were examined. lv, liver tissue; we, wound epidermis. Scale bars: 75 µm (*A,B*) and 100 µm (*C*).

During the wound healing process, fibroblast cells were expected to migrate to the amputation site and then proliferate to form a stratified epithelialised cell mass called the wound epidermis. Following immunostaining with keratin 18, an extracellular matrix marker produced by the epithelial cells [20], we found that the new Bhmt^−^ cells at the amputation site in both wild-type and *def^+/−^* fish at 3 days after PH (Figure 3C) expressed high levels of keratin 18 (Figure 4A), and that the new cell mass we observed (Figure 3C) therefore actually represented the wound epidermis at the amputation site after PH.

**Figure 5:**
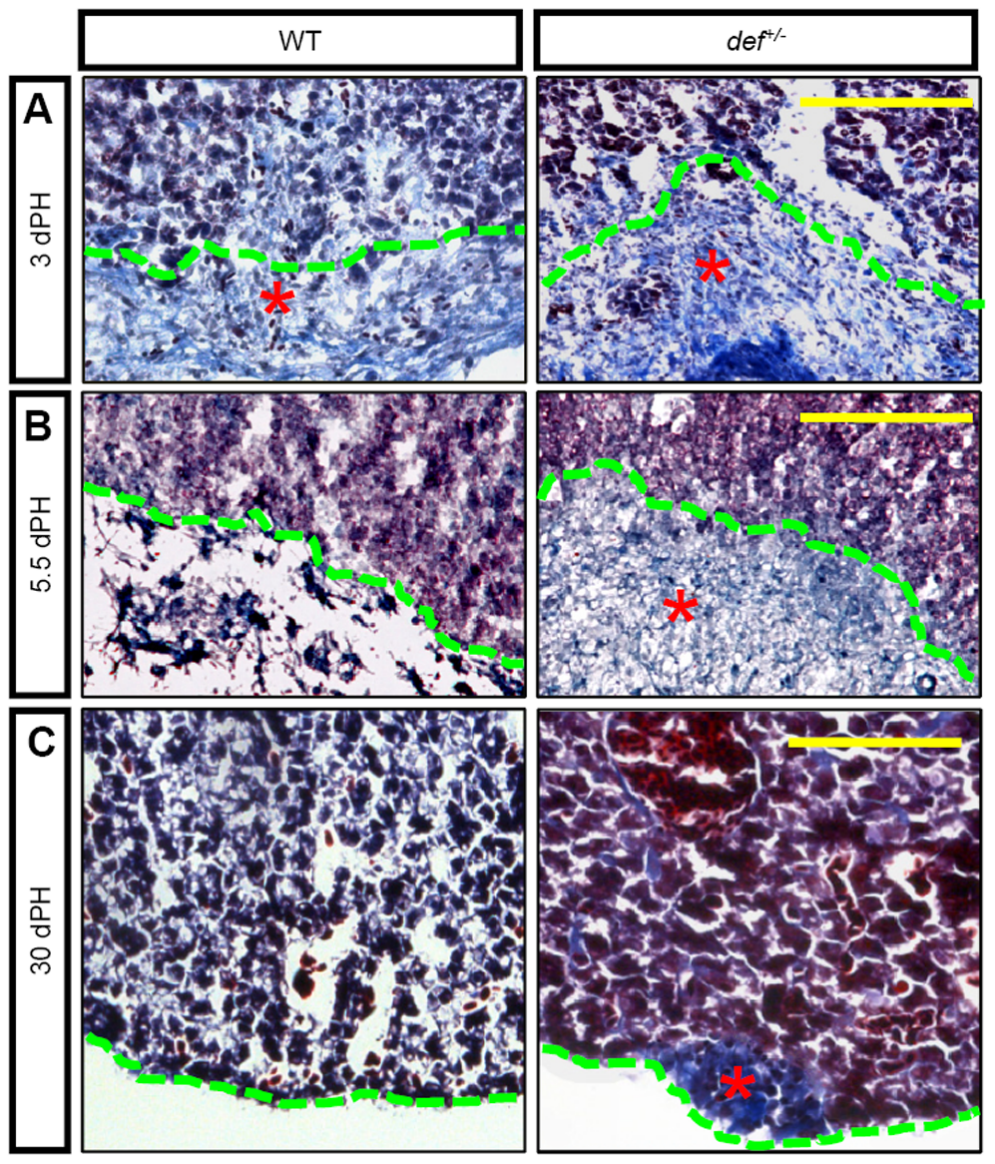
Def haploinsufficiency results in the formation of a fibrotic scar at the amputation site after PH. Masson staining of sections across the amputation site 3 (*A*), 5.5 (*B*) and 30 (*C*) days after PH (dPH), respectively. Fibrotic tissue was stained in blue (highlighted with an asterisk), cytoplasm in light red and nuclei in dark brown. Green, dashed lines outline the amputation site. Scale bar: 100 µm.

To determine the difference between the wound epidermis of the wild-type and *def^+/−^* fish, we performed immunostaining with type I collagen (ColI), a marker for fibrotic tissue [21]. The results showed that the wound epidermis (keratin 18^+^ cells) in the wild-type liver displayed weak ColI expression whereas that in *def^+/−^* produced large amounts of ColI (Figure 4B; Figure S1C) 3 days after PH, which was further confirmed by Masson staining (the collagen was stained blue) (Figure 5A). By 5.5 days after PH, Masson staining showed that the difference in collagen staining between the amputation site and the liver tissue in the wild-type fish was no longer distinguishable, whereas the wound epidermis in the *def^+/−^*fish was still clearly visible and strongly expressed collagens (Figure 5B). The wound epidermis in the wild-type fish was successfully remodelled to a proper liver structure by 30 days after PH (Figure 5C). In contrast, the wound epidermis in the *def^+/−^* fish eventually formed a fibrotic scar characterized by the strong staining of collagens at the amputation site (Figure 5C).

### Activation of TGFβ Signalling Is Responsible for Collagen Deposition At the Amputation Site after PH in *def^+/−^* Fish

**Figure 6:**
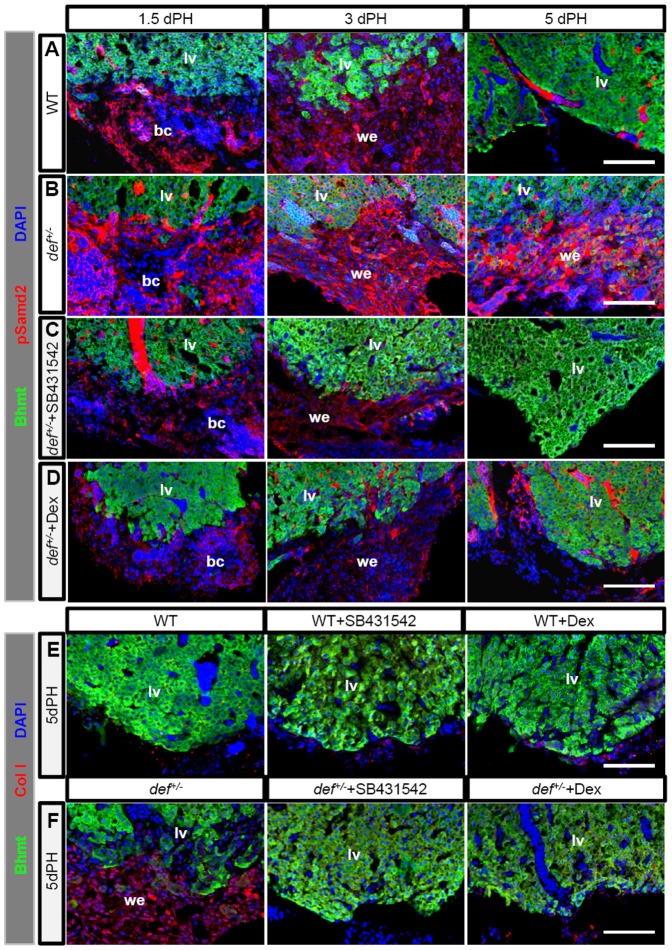
Constitutive activation of TGFβ signalling promotes ColI deposition at the amputation site in *def^+/−^* fish after PH. (*A–D*) Representative images of immunostaining of pSmad2 (red) and Bhmt (green) in wound epidermis in wild-type (*A*), *def^+/−^* (*B*), *def^+/−^* treated with SB431542 (*C*) or *def^+/−^*treated with Dex (*D*) 1.5, 3 and 5 days after PH, respectively. (*E,F*) Representative images of immunostaining of ColI (red) and Bhmt (green) in the wound epidermis in wild-type and *def^+/−^*fish treated or not treated with SB431542 (*E*) or Dex (*F*) 5 days after PH, respectively. Nuclei were stained with DAPI (blue). In each case, more than 10 sections from at least three wild-type or *def^+/−^* mutant fish were examined. bc, blood clot; lv, liver tissue; we, wound epidermis. Scale bar:75 µm (*A–F*).

ColI production is closely related to TGFβ signalling activity [22]. We next examined the status of phosphorylated Smad2 (pSmad2), a hallmark for the activation of TGFβ signalling [23]. At 1.5 and 3 days after PH, the wound epidermis in both the wild-type and *def^+/−^* fish showed positive pSmad2 staining, with *def^+/−^* exhibiting a stronger signal intensity (Figure 6A,B). At 5 days after PH, the pSmad2 signal disappeared together with the wound epidermis in the wild-type strain (Figure 6A). Strikingly, in the *def^+/−^* strain, the wound epidermis failed to be remodelled, with persistent, strong pSmad2 staining at the amputation site (Figure 6B; Figure S5A). To prove that the constitutive activation of TGFβ signalling is responsible for the ColI deposition in *def^+/−^*, we injected SB431542 (Alk5/4-i) intraperitoneally into the amputated fish. SB431542 specifically blocks the TGFβ/activin pathway receptors but does not affect closely related receptors from the bone morphogenetic protein pathway or other signal transduction pathways [24], [25]. The results showed that this treatment efficiently down-regulated the expression level of pSmad2 (Figure 6C; Figure S5A) and abolished ColI deposition at the amputation site in the liver of *def^+/−^*fish5 days after PH (Figure 6E and F; Figure S5B).

### An Abnormal Inflammatory Reaction Triggers An Increase in TGFβ Signalling in *def^+/−^* Fish

We sought the mechanism(s) that activate(s) TGFβ signalling in the wound epidermis. We first treated the fish with two chemicals, diphenyleneiodonium and apocynin (both of which are specific inhibitors of the Duox/Nox enzyme [26]) to down-regulate the production of hydrogen peroxide, which has been shown to be related to the fibrotic scar formation during heart regeneration (Jingwei Xiong, personal communication). We found that neither of these two chemicals induced ColI deposition in the liver of the wild-type fish or inhibited its deposition in the liver of the *def^+/−^* fish at the amputation site after PH (Figure S6), indicating that the deposition of collagens in this case was independent of the reactive oxygen species signalling pathway. Fibrotic scar formation closely correlates with prolonged local inflammatory reaction. Dexamethasone (Dex) is a potent synthetic member of the glucocorticoid class of steroid drugs that has anti-inflammatory and immunosuppressant properties [27]. Treatment with Dex dramatically down-regulated the expression of pSmad2 in the wound epidermis (Figure 6D; Figure S5A) and efficiently blocked the deposition of ColI at the amputation site in *def^+/−^*fish after PH (Figure 6E and F; Figure S5B).

### Abnormal Expression of Cytokines in the *def^+/−^* Liver Halted the Timely Migration of Macrophages To the Amputation Site To Clear Neutrophils

**Figure 7:**
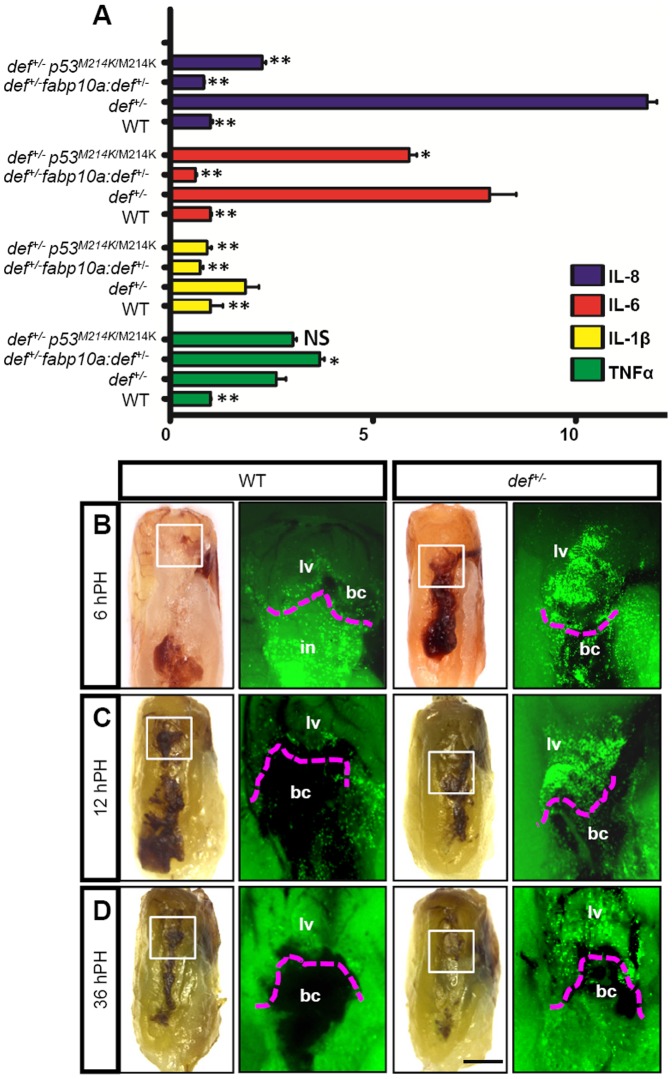
*def^+/−^* mutant liver exhibited a prolonged inflammatory reaction at the amputation site after PH. (*A*) qPCR analysis of cytokine genes *TNFα*, *IL-1β*, *IL-6* and *IL-8* in adult wild-type, *def^+/−^, fabp10a:def^+/−^* and *def^+/−^ p53^M214K^* ^/M214K^ livers prior to the surgical procedure. Gene expression is expressed as the fold change after normalisation against *β*-*actin*. The value for each gene in the wild-type was set at 1. The *p* value represents the statistical differences between *def^+/−^* and another corresponding group. *: *p*<0.05, **: *p*<0.01, NS: not significant. (*B–D*) Adult fish of *Tg(zlyz:EGFP)* in a wild-type and *def^+/−^* background were subjected to PH, and collected 6 h after PH (*B*), 12 h after PH (*C*) and 36 h after PH (*D*) for EGFP fluorescence imaging. Images were taken from the region outlined in white in the corresponding bright field picture shown on the left. The region above the dashed magenta lines is the liver tissue adjacent to the amputation site. Black asterisks highlight the EGFP signal yielded possibly from neutrophils in the intestine. A representative image is shown for each time-point. bc, blood clot; in, intestine; lv, liver tissue. Scale bar: 5 mm (bright field) and 2 mm (GFP field).

The previous result suggested that an abnormal inflammatory response is probably the main mechanism that activates TGFβ signalling and causes scar formation in the *def^+/−^* liver. To test this hypothesis, we first analysed the expression of pro-inflammatory cytokines, including *tumour necrosis factor α* (*TNFα*), *IL-1β*, *IL-6* and *IL-8* [28]–[30], in the livers of wild-type and *def^+/−^* fish prior to surgical operation. We found that *IL-6*, *IL-8* and *TNFα* were expressed at levels several fold higher and *IL-1β* at a significantly higher level in the liver of the *def^+/−^*than in that of the wild-type fish (Figure 7A), which suggested that the innate immune response was probably affected in the *def^+/−^* fish after acute injury (i.e., after PH).

**Figure 8:**
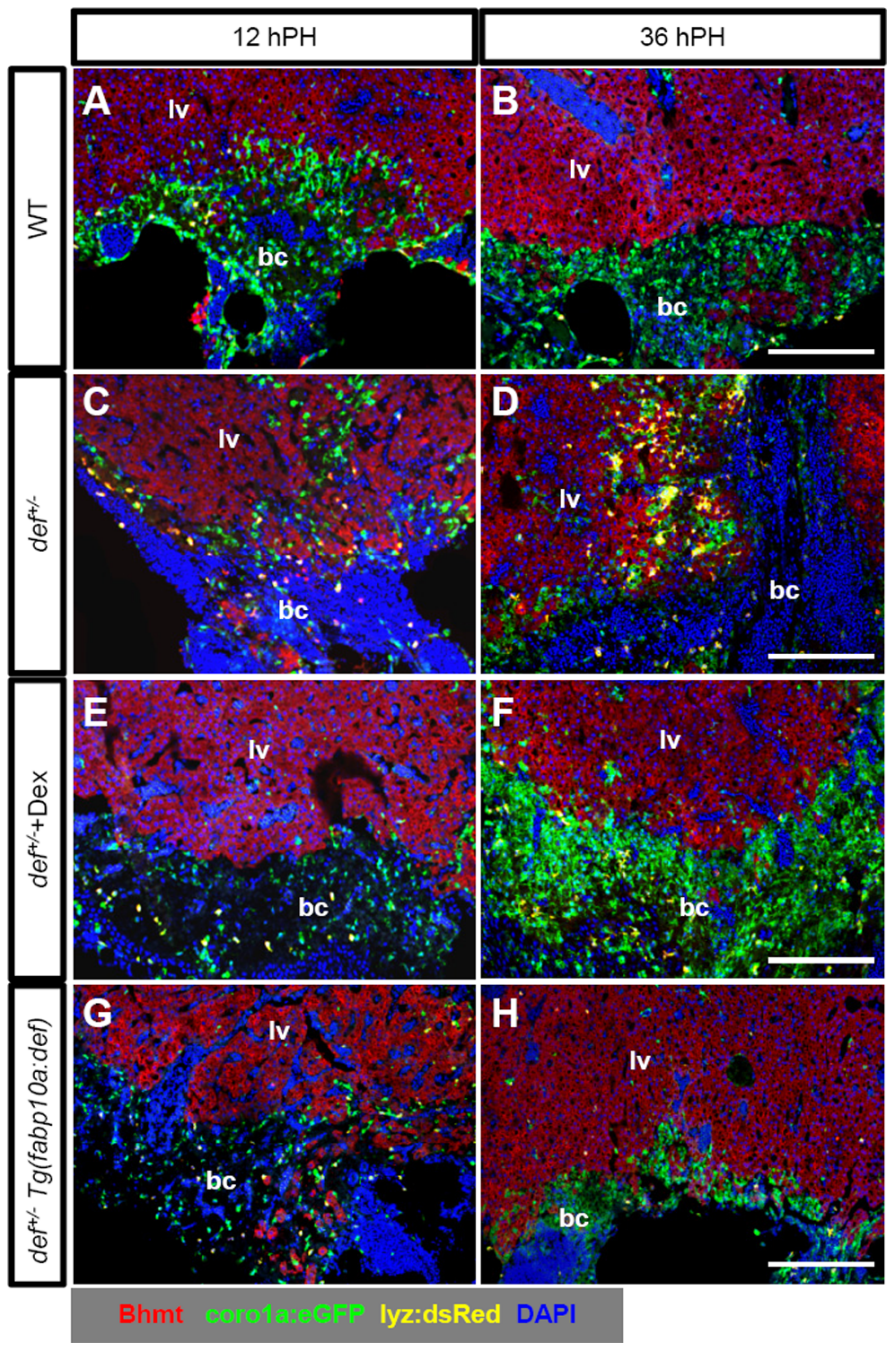
Def haploinsufficiency caused an altered migration behaviour of leukocytes in *def^+/−^* fish that was rescued by treatment with Dex or ectopic expression of Def in hepatocytes. (*A–H*) Representative images of co-immunostaining of EGFP (green, label macrophage), dsRed (yellow, label neutrophil) and Bhmt (red) in the wound epidermis in wild-type (*A,B*), *def^+/−^* (*C,D*), *def^+/−^* treated with Dex (*E,F*) and *def^+/−^Tg(fabp10a:def)* progeny (*G,H*) 12 and 36 h after PH, respectively. All are in the background of *Tg(coro1a:eGFP;lyz:dsRED)*. Nuclei were stained with DAPI (blue). In each case, more than 10 sections from at least three wild-type or *def^+/−^* mutant fish were examined. bc, blood clot; lv, liver tissue. Scale bars: 150 µm (*A–H*).

We then used the *Tg(zlyz:EGFP)* reporter fish to monitor the inflammatory event by observing the dynamics of neutrophils at the amputation site. We noted that the number of neutrophils that accumulated at the amputation site in the *def^+/−^* fish was much greater than that in the wild-type fish between 6 and 36 h after PH (Figure 7B–D). In addition, while only a few neutrophils remained in the wild-type, a large number of neutrophils still lingered around the amputation site in the *def^+/−^* fish at 36 hours after PH (Figure 7D), suggesting the amputation site in the *def^+/−^* fish likely suffered from a prolonged inflammatory reaction due to the delayed clearance of neutrophils by macrophages. This prompted us to examine the behaviour of macrophages using a double-labelled transgenic fish,*Tg(coro1a:eGFP;lyz:dsRed)*. In this reporter fish, both macrophages and neutrophils are genetically labelled by enhanced green fluorescent protein (EGFP) while neutrophils are also labelled by dsRed [31]. We surprisingly found that the number of macrophages at the amputation site was much less in the *def^+/−^* than that in the wild-type fish at 12 and 36 h after PH. This observation effectively explained why the clearance of neutrophils at the injured site was severely delayed (Figure 8A–D; Figure S7). Treatment with Dex restored the behaviour of the macrophages (Figure 8E,F; Figure S7), demonstrating that the process of wound healing closely correlates with the resolution of inflammation *in situ* in a regenerating liver.

### Hepatic Haploinsufficiency of Def Causes Scar Formation in *def^+/−^* Fish

**Figure 9:**
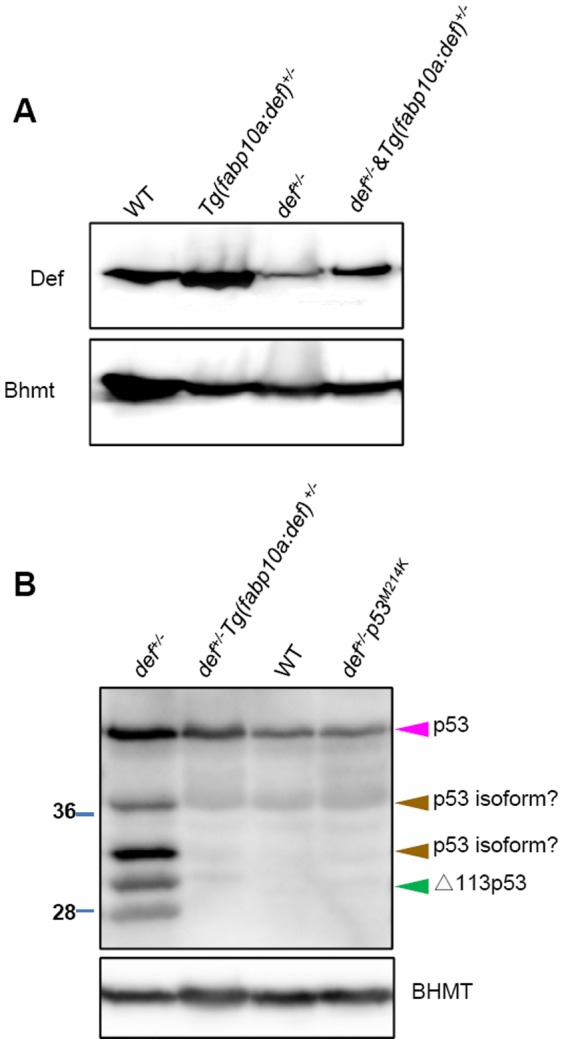
Def expression in the *def^+/−^* hepatocytes was restored in the *def^+/−^ Tg(fabp10a:def)* fish and Def haploinsufficiency up-regulates p53 and Δ113p53 expression in the *def^+/−^* liver. (*A*) Western blot of Def using an antibody against zebrafish Def to compare its expression levels in the wild-type, *Tg(fabp10a:def)*, *def^+/−^* and *def^+/−^Tg(fabp10a:def)* adult livers. Bhmt: loading control. (*B*) Western blot of p53 and Δ113p53 in the wild-type, *Tg(fabp10a:def)*, *def^+/−^* and *def^+/−^p53^M214K/M214K^* adult livers using an antibody against zebrafish p53. Two uncharacterised p53 isoforms are highlighted by a brown arrow head. Bhmt: loading control.

**Figure 10:**
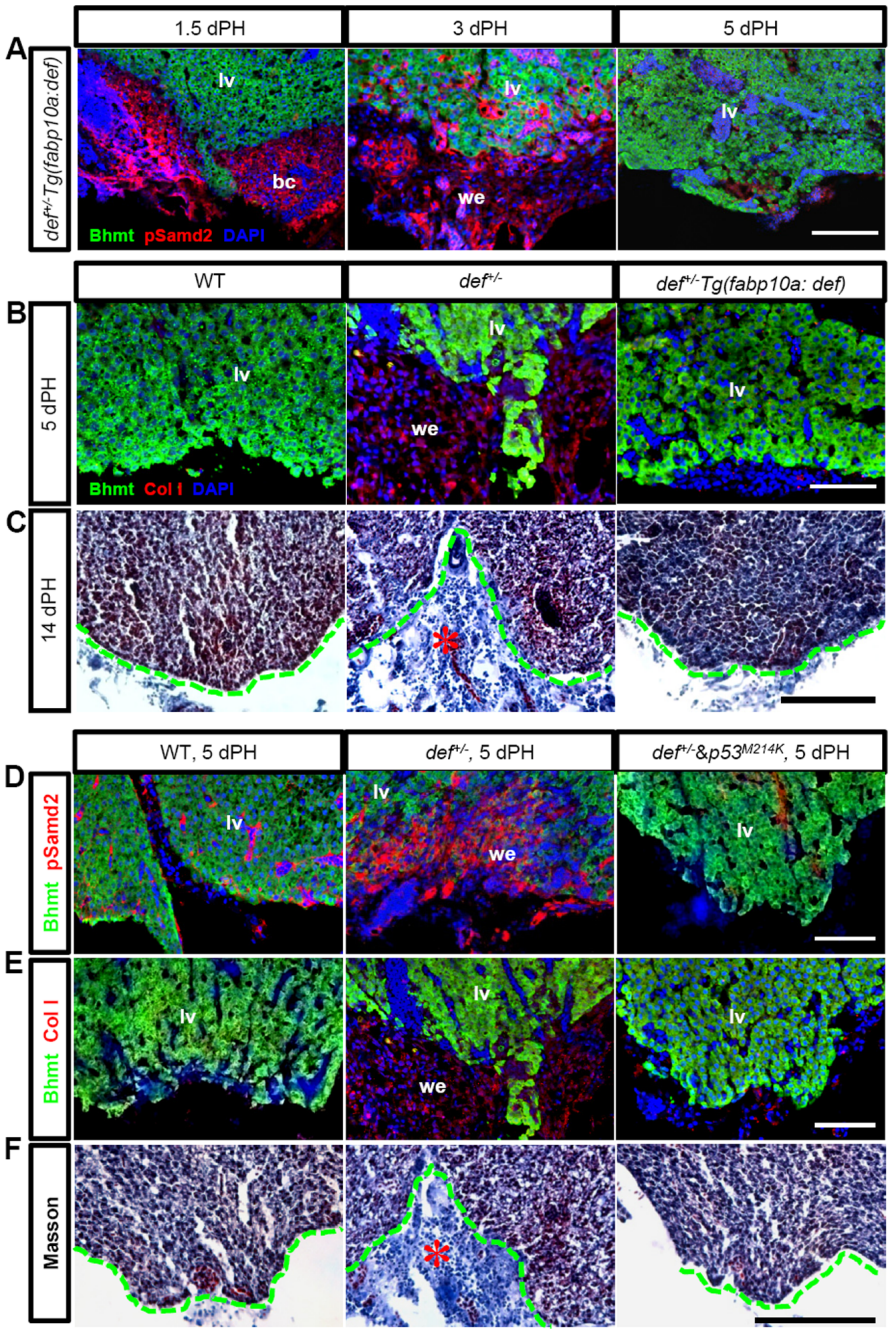
Formation of the fibrotic scar in *def^+/−^* fish after PH was mediated by the activation of the p53-pathway. (*A*) Immunostaining of pSmad2 (red) and Bhmt (green) in the wound dermis in *def^+/−^Tg(fabp10:def)^+/−^* progeny 1.5, 3 and 5 days after PH, respectively. (*B*) Immunostaining of ColI (red) and Bhmt (green) in the wild-type, *def^+/−^* and *def^+/−^Tg(fabp10:def)^+/−^* progeny 5 days after PH. (*C*) Masson staining of the amputation site (indicated by a green dashed line) in the wild-type, *def^+/−^* and *def^+/−^ Tg(fabp10:def)^+/−^* progeny 14 days after PH. (*D,E*) Immunostaining of pSmad2 (red) and Bhmt (green) (*D*) and of ColI (red) and Bhmt (green) (*E*) in the wound epidermis in the wild-type, *def^+/−^* and *def^+/−^p53^M214K/M214K^* 5 days after PH. (*F*) Masson staining of the amputation site in the wild-type, *def^+/−^* and *def^+/−^p53^M214K/M214K^* progeny 14 days after PH. Fibrotic tissue in the *def^+/−^* fish is highlighted by a red asterisk. In (*A,B,D,E*), nuclei were stained with DAPI (blue). In each case, more than 10 sections from at least three wild-type or *def^+/−^* mutant fish were examined. Representative images are shown here. bc, blood clot; lv, liver tissue; we, wound epidermis. Scale bars: 150 µm (*C,F*) and 75 µm (*A,B,D,E*).

To determine the role of hepatic Def in the formation of fibrotic scars, we crossed *def^+/−^* mutants with a transgenic line (*Tg(fabp10:def)*), in which *def* expression is driven by the promoter of a liver-specific gene *fabp10* [32]. Def expression in the *def^+/−^Tg(fabp10a:def)* progeny was restored to that found in the wild-type fish (Figure 9A). Immunostaining of pSmad2 showed that the aberrantly high level of pSmad2 was almost completely restored to normal in *def^+/−^Tg(fabp10a:def)* progeny at 5 days after PH (Figure 10A; Figure S5A). Immunostaining showed that the amount of ColI deposited at the amputation site in the *def^+/−^Tg(fabp10:def)^+/−^* progeny was down-regulated to a level comparable with that in wild-type fish at 5 days after PH (Figure 10B; Figure S5C). Hepatic over-expression of Def in the *Tg(fabp10a:def)* fish also partially restored the behaviour of macrophages in the *def^+/−^* strain (Figure 8G–H; Figure S7), which correlated with the down-regulation of *IL-1β*, *IL-6* and *IL-8* in the *def^+/−^Tg(fabp10:def)^+/−^* fish (Figure 7A). Furthermore, Masson staining showed that the amputation site was successfully remodelled to liver tissue by 14 days after PH (Figure 10C). These results demonstrate that Def haploinsufficiency in hepatocytes is the causative agent of fibrotic scar formation in *def^+/−^* fish.

### Loss of Function of p53 Suppresses Scar Formation in *def^+/−^* Fish after PH

We recently showed that Def complexes with Capn3 to target p53 for degradation in the nucleolus [14]. We first examined the level of p53 and △113p53 in *def^+/−^* fish and found that their expression was up-regulated in the adult *def^+/−^* liver, and that restoration of the Def protein level in the hepatocytes by *Tg(fabp10a:def)* reduced the p53 and △113p53 expression in *def^+/−^* to a level similar to that exhibited by the wild-type fish (Figure 9B). We then investigated whether constant activation of p53 in the *def^+/−^* fish promoted inflammation and caused fibrotic scar formation after PH. We crossed the *def^+/−^* mutant with a *p53^M214K^* mutant to obtain *def^+/−^p53^M214K/M214K^* progeny for PH. Immunostaining analysis revealed that the loss of function of p53 significantly down-regulated the level of pSmad2 (Figure 10D) and inhibited ColI deposition at the amputation site in the *def^+/−^* fish (Figure 10E; Figure S5C) at 5 days after PH. Moreover, Masson staining also showed that the wound site was completely remodelled without leaving a fibrotic scar at 14 days after PH (Figure 10F). Interestingly, quantitative polymerase chain reaction (qPCR) analysis showed that only the expression of *IL-1β* and *IL-8* but not that of *IL-6* or *TNFα* was down-regulated to a level closer to that found in the wild-type in the *def^+/−^p53^M214K/M214K^* liver (Figure 7A), suggesting that Def regulates the expression of *IL-6* and *TNFα*, probably through other signalling pathways.

### p53 Activation in *def^+/−^* Fish Up-regulates the Expression of the Pro-inflammatory Factor HMGB1

**Figure 11:**
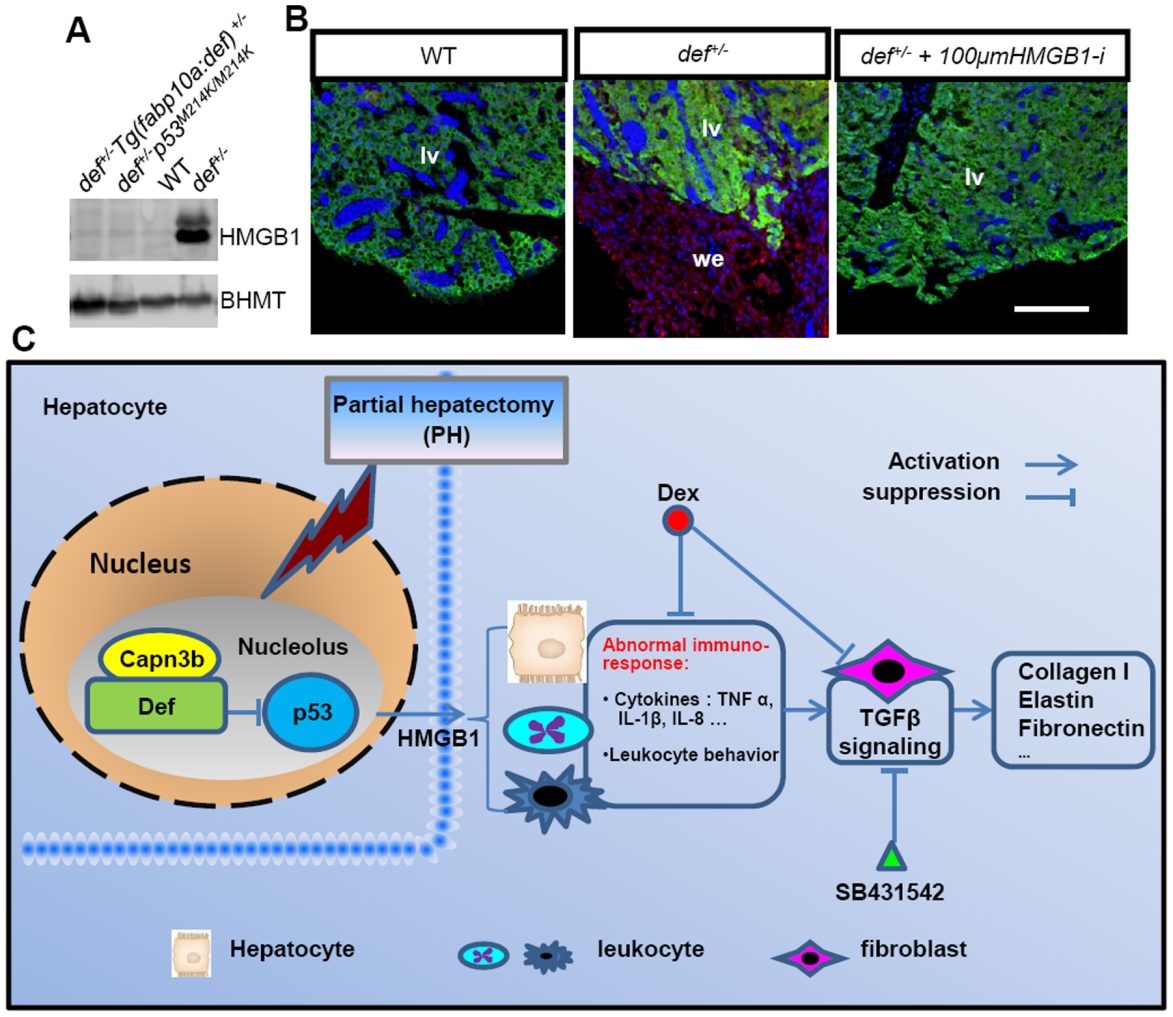
A model to explain how Def haploinsufficiency activates the p53-dependent inflammation-mediated TGFβ signalling and causes fibrotic scar formation in *def^+/−^* after PH. (*A*) Western blot of HMGB1 in the adult liver from wild-type, *def^+/−^*, *def^+/−^Tg(fabp10:def)^+/−^* and *def^+/−^p53^M214K/M214K^* fish. Loading control: Bhmt. (*B*) Immunostaining of ColI and Bhmt in the wound epidermis in wild-type, *def^+/−^* and *def^+/−^*fish treated with 100 µM of glycyrrhizic acid ammonium 5 days after PH. lv, liver; we, wound epidermis. (*C*) In a wild-type liver, Def complexes with Capn3 to mediate p53 degradation in the nucleoli to mitigate the inflammatory response after acute injury. In *def^+/−^* fish, the activity of the Def-Capn3 protein degradation pathway is compromised and p53 protein is thus stabilised, which in turn activates the p53 pathway. Constant activation of the p53 pathway up-regulates the expression of the pro-inflammatory factor HMGB1 that up-regulates the expression of cytokines (e.g., TNFα, IL-1β, IL-6 and IL-8) and other signalling pathways that lead to a prolonged inflammatory response in *def^+/-^*. The prolonged inflammatory response activates TGFβ signalling and causes the over-production of fibrotic molecules (e.g., collagens, elastin and fibronectin) in the wound epidermis that finally forms a fibrotic scar at the amputation site in *def^+/−^*. Fibrotic scar formation in *def^+/−^* can be blocked by over-expressing Def specifically in the hepatocytes, by the loss of function of p53, and by treatment with Dex (anti-inflammatory drug), SB431542 (inhibitor of TGFβ signalling) or glycyrrhizic acid ammonium (inhibitor of HMGB1 function).

HMGB1 is a dual function protein that serves either as a nuclear factor that can loosely bind to chromatin or as an extracellular molecule to promote the inflammatory response [33], [34]. A recent report showed that p53 activation induces the release of HMGB1 protein to promote inflammation-associated hepatocarcinogenesis [35]. We first compared the expression of HMGB1 in the adult livers from wild-type, *def^+/−^*, *def^+/−^Tg(fabp10a:def)* and *def^+/−^p53^M214K/M214K^* fish, and found that the level of HMGB1 protein was highly elevated in the *def^+/−^* fish but was significantly down-regulated to the level found in the wild-type in both the *def^+/−^Tg(fabp:def)* and *def^+/−^p^214K/M214K^* strains (Figure 11A). We then treated the amputated *def^+/−^* fish with glycyrrhizic acid ammonium, an inhibitor of HMGB1, and found that it blocked the deposition of ColI at the amputation site(Figure 11B; Figure S5C).

## Discussion

Immediately after injury, extravasated blood constituents form a haemostatic plug. Platelets and neutrophils entrapped in the blood clot release a wide variety of factors that serve as chemoattractants to guide the migration of leukocytes, including firstly neutrophils followed by macrophages. Macrophage infiltration into the wound site is responsible for the clearance of neutrophils to resolve local inflammation [36], [37]. We found that neutrophil clearance from the amputation site was delayed in *def^+/−^* fish due to the failure of macrophages to migrate to the injured site in a timely fashion. We believe that this was probably due to disruption of the cytokine gradient because *TNFα*, *IL-1β*, *IL-6* and *IL-8*, four cytokine genes known to modulate leukocyte responses [30], were highly expressed in the *def^+/−^* liver prior to surgical operation. The prolonged inflammation in the *def^+/−^* fish consequently activated TGFβ signalling in the wound epidermis that finally resulted in fibrotic scar formation after PH. This conclusion was further confirmed by the fact that treatment with both Dex and SB431542 significantly blocked fibrosis at the amputation site.

Interestingly, we found that the defect (i.e., fibrotic scar formation) in wound healing after PH in the *def^+/−^* fish was caused by the haploinsufficiency of Def in the hepatocytes, because the restoration of Def expression in the *def^+/−^* hepatocytes rescued the healing of the amputation site in the *def^+/−^* fish after PH. We then questioned what role Def, a nucleolar factor, plays in the wound healing process after PH. We recently demonstrated that Def complexes with Capn3 to mediate p53 degradation in the nucleolus and that the level of p53 protein was highly elevated in the *def^−/−^* homozygous mutant [14]. Here, we show that the loss of function of p53 in the *def^+/−^* background down-regulated the expression of *IL-1β* and *IL-8* and suppressed fibrotic scar formation after PH. This genetic evidence not only demonstrated unequivocally that the activated p53 pathway contributes to the aberrant expression of cytokines in the *def^+/−^* liver but also suggested that *IL-1β* and *IL-8* are probably the key players in this process. A recent report showed that constant activation of p53 promotes inflammation by inducing the release of HMGB1 from hepatocytes [35]. Indeed, we found that the expression of HMGB1 was elevated in the *def^+/−^* liver and that inhibition of the HMGB1 function by the administration of glycyrrhizic acid ammonium blocked the deposition of ColI at the amputation site.

Based on the data available, we propose that the Def-Capn3-p53 pathway negatively affects the inflammatory response in the liver, probably through the regulation of HMGB1 expression. Disruption of this pathway leads to the constant activation of p53 to promote the inflammatory response in the liver that in turn alters the balance between the recruitment and clearance of leukocytes around the amputation site over time after PH, and consequently causes fibrotic scar formation at the amputation site (Figure 11C). However, some key questions remain unanswered. For example, how exactly does p53 regulate the expression of HMGB1? Are other signalling pathways involved in the remodelling of the wound epidermis? The answers to such questions will no doubt help to unravel the mystery of scarless healing of the wound during liver regeneration after PH.

## Materials and Methods

### Ethics Statement

All animal procedures were performed in full accordance to the requirement by ‘Regulation for the Use of Experimental Animals in Zhejiang Province'. This work is specifically approved by the Animal Ethics Committee in the School of Medicine, Zhejiang University (ETHICS CODE Permit NO. ZJU2011-1-11-009Y, issued by the Animal Ethics Committee in the School of Medicine, Zhejiang University).

### Zebrafish Strains

The two pairs of primers used for genotyping *def^hi429^* mutants have been described previously [13]. The *Tg(fabp10a:dsRed)* transgenic line was used to label the hepatocytes [38], [39], the *Tg(zlyz:EGFP)* transgenic line was used to label the neutrophils [40] and the *Tg(coro1a:eGFP;lyz:dsRed)* transgenic line was used to label macrophages (green) and neutrophils (red), respectively [31]. The *p53^M214K^* mutant was provided by Prof. Thomas Look [41]. The transgenic and the mutant lines were crossed with a *def^+/−^* heterozygous mutant.

### Zebrafish Manipulation and Measurement of the Liver-versus-Body Ratio

All of the zebrafish used for PH in this study were between 5 and 7 months of age. The PH operation and measurement of the liver-versus-body ratio were performed as described previously [9]. The survival rates of the wild-type and *def^+/−^* fish after surgery were greater than 95% up to 60 days after PH in all of the experiments described in this work. For drug treatment, 20 µM diphenyleneiodonium (D2926; Sigma) or 200 µM apocynin (W508454; Sigma) was injected into the abdominal cavity daily between days 0 and 2 after PH; 20 mg/L of Dex (D4902; Sigma) was injected immediately after PH; 100 µM of glycyrrhizic acid ammonium salt (50531; Sigma) was injected daily between days 0 and 3 after PH; and 20 µM of SB431542 (ITB1001; Gene Operation) was injected 2 days after PH.

### Histological Analysis

Immunohistochemistry was performed as described previously [13]. The antibodies used in this study were mouse anti-PCNA (P8825; Sigma), rabbit anti-red fluorescent protein (ab34771; Abcam), rabbit anti-Collagen I (ab34710; Abcam), rabbit anti-pSmad2 (3101S; Cell Signalling), guinea-pig anti-keratin18 (LS-C22655; Lifespan), chicken anti-GFP (ab13970; Abcam), mouse anti-p53 [14], rabbit anti-Def [13], mouse anti-Bhmt [17] and rabbit anti-HMGB1 (ab18256). The anti-rabbit, -mouse, -guinea, -pig and -chicken secondary antibodies (Invitrogen) were conjugated with Alexa Fluor 647, 568, 633 and 488, respectively. The TUNEL assay was carried out using the In Situ Cell Death Detection Kit, TMR red (Roche). All images were taken under a Leica TCS SP5 microscope or an Olympus FV-ASW confocal microscope.

For the EdU incorporation assay, the fish were anaesthetised in 0.02% Tricaine, and 10 µl of EdU (125 mg/ml in dimethyl sulphoxide solution) was injected into the abdominal cavity immediately after PH followed by another injection 24 h after PH. The fish were then collected and fixed for cryosectioning. EdU staining was performed using an EdU Imaging Kit (Life Technologies). Masson staining was performed according to the protocols described at http://www.ihcworld.com/. All images were taken under a Nikon AZ100 microscope.

### Protein and RNA Analysis

Total protein extraction and western blotting were carried out as described previously [42]. The adult zebrafish liver was treated in cell lysis buffer (63 mM of Tris-HCl, 10% glycerol, 5% β-mercaptoethanol, 3.5% sodium dodecyl sulfate) in the presence of a protease inhibitor, Complete (11873580001; Roche). Total RNA extraction and qPCR were performed on a Bio-Rad Real-Time System using the fluorescence-labelled dNTP mix (Bio-Rad) according to the manufacturer's instructions. The experiments were performed in triplicate. The sequences of the primer pairs used for qPCR were as follows: *TNFα* forward primer: GTTTATCAGACAACCGTGGCCA, reverse primer: GATGTTCTCTGTTGGGTTTCTGAC; *il-1β* forward primer: TGGACTTCGCAGCACAAAATG, reverse primer: CACTTCACGCTCTTGGATGA; *il-6* forward primer:TCAACTTCTCCAGCGTGATG,reverse primer:TCTTTCCCTCTTTTCCTCCTG, *il-8* forward primer:GTCGCTGCATTGAAACAGAA, reverse primer:CTTAACCCATGGAGCAGAGG.

## Supporting Information

Figure S1 **Statistic analysis of the signal intensity of the immunostaining for Def, PCNA and ColI, respectively.** (*A*) Comparison of Def signal intensities among WT sham, *def^+/−^* sham, WT and *def^+/−^* at 2 dPH as showing in Figure 1C. The relative value of the Def signal intensity in *def^+/−^* was set at 1. (*B*) Comparison of the index of proliferating cells at the amputation site at 3 dPH as showing Figure 3C. Hepatocytes (Bhmt^+^ cells) and wound epidermal cells (Bhmt^−^ cells) in the same areas at the amputation plane were counted. PCNA+ hepatocytes and wound epidermal cells were also counted. The average ratio of total PCNA^+^ hepatocytes (PH) to total hepatocytes (TH) and total PCNA^+^ epidermal cells (PEC) to total wound epidermal cells (TEC) in WT and *def^+/−^* were obtained, respectively. The value of PEC/TEC in *def^+/−^* was set as 1 for the convenience of comparison. Data were collected from three fish for each genotype in each case. (C) Comparison of the ColI signal intensities at the amputation site between WT and *def^+/−^* at 3dPH as showing in Figure 4B. The relative value of the ColI signal intensity in WT was set as 1. Ten sections from three WT or *def^+/−^* mutant fish were examined. In (*A–C*), the values plotted represent the means ± standard errors of the mean. The *p*-value was obtained by performing the two-tailed unpaired *t*-test. *** P<0.001; Student's *t* test. Signal intensity in each case was acquired by Photoshop based on the brightness of immunostaining of the targeted protein in a selected region.
(TIF)

Figure S2 **The** ***def^+/−^*** **mutant showed defective lobe structure recovery at the amputation site after PH.** Lower magnifications of the ventral and lateral views of the gross morphology of the liver 3, 7 and 21 days after PH. The images for 7 days after PH correspond to the closer view of the amputation site shown in Figure 1d. The white, dashed line outlines the amputation site on the ventral tip. The red arrow highlights the ventral lobe in the sham control. VL: ventral lobe; LDL: left dorsal lobe; RDL: right dorsal lobe. Scale bar: 1 cm.
(TIF)

Figure S3 **Comparison of the wound healing process between the wild-type and** ***def^+/−^*** **livers after PH.** Frontal plane sections of the liver 12, 24 and 36 h after PH were stained for apoptotic cells (in white, using the TUNEL assay), proliferative cells (in red, by EdU incorporation) together with Bhmt (hepatocyte marker) (in green). DAPI was used to stain the nuclei (blue). All images show the part of the hepatic tissue adjacent to the amputation site. Yellow dashed lines outline the amputation site, while regions defined by red brackets represent the blood clot. (*A–D*) The blood clot was clearly formed in both the wild-type (*A*) and *def^+/−^* mutant (*B*) fish 12 h after PH. Cells in the blood clot underwent massive apoptosis 24 h after PH (*C,D*). (*E,F*) By 36 h after PH, the blood clot was detached from the liver mass in the wild-type fish (*E*), whereas it was still connected to the liver mass in the *def^+/−^* mutant (*F*). The inset, enlarged view corresponds to the amputation site (*A–F*). bc, blood clot; lv, liver tissue. Scale bar: 250 µm (*A–F*).
(TIF)

Figure S4 **Comparison of cell proliferation in the adult liver in the wild-type and** ***def^+/−^*** **sham controls.** Representative images of immunostaining of PCNA (in red) in the *Tg(fabp10a:RFP)* background, in which hepatocytes are genetically labelled by expressing the red fluorescent protein (in green), showed that proliferating cells were rarely detected along the epithelial edge of an adult liver in either the wild-type or *def^+/−^* mutant sham controls. Nuclei were stained with DAPI (blue). lv, liver tissue. Scale bar: 75 µm.
(TIF)

Figure S5 **Statistic analysis of the effect of different treatment on the signal intensity of the immunostaining for pSmad2 and ColI at the amputation site, respectively.** (*A*). Comparison of the pSmad2 signal intensities in WT, *def^+/−^*, *def^+/−^* treated with SB431542 or Dex, and *def^+/−^ Tg(fabp10a:def)* at 1.5, 3 and 5dPH, respectively, as showing in Figure 6A–D and Figure 10A. Ten sections from three fish for each genotype in each case were examined. (*B*) Comparison of the ColI signal intensities in WT, *def^+/−^*, WT or *def^+/−^* treated with SB431542 or Dex at 5dPH as showing in Figure 6E and 6F. Ten sections from three fish in each case were examined. (*C*) Comparison of the ColI signal intensities in WT, *def^+/−^*, *def^+/−^ Tg(fabp10a:def), def^+/−^p53^M214K/M214K^* or *def^+/−^* treated with HMGB1-i at 5dPH as showing in Figure 10B and 10E and Figure 11B. Ten sections from three fish for each genotype in each case were examined. In (*A–C*), the values plotted represent the means ± standard errors of the mean. The *p*-value was obtained by performing the two-tailed unpaired *t*-test. *** P<0.001; NS: non significant. Signal intensity in each case was acquired by Photoshop based on the brightness of immunostaining of the targeted protein in a selected region.
(TIF)

Figure S6 **ColI deposition at the amputation site in** ***def^+/−^*** **is not mediated by the reactive oxygen species signalling pathway.** Representative images of co-immunostaining of ColI (red) and Bhmt (green) in the wound epidermis in wild-type, *def^+/−^* and *def^+/−^* fish treated with diphenyleneiodonium (DPI) or apocynin (APO), two inhibitors of the reactive oxygen species signalling, 5 days after PH. Nuclei were stained with DAPI (blue). lv, liver tissue; we, wound epidermis. Scale bar: 75 µm.
(TIF)

Figure S7 **Comparison of the ratio of macrophages to neutrophils at the amputation site.** The number of macrophages and neutrophils at the amputation site were counted, respectively, in WT, *def^+/−^*, *def^+/−^* treated with Dex, and in *def^+/−^ Tg(fabp10a:def)* at 12 and 36 hPH as showing Figure 8. Ten sections from three fish in each case were examined and the ratio of macrophages to neutrophils was obtained for each section to minimize the variation caused by sectioning. The values plotted represent the means ± standard errors of the mean. The *p*-value was obtained by performing the two-tailed unpaired *t*-test. * P<0.05,** P<0.01. Signal intensity in each case was acquired by Photoshop based on the brightness of immunostaining of the targeted protein in a selected region.
(TIF)
