# Supplementary figures and images for: Haploinsufficiency of Def Activates p53-Dependent TGFβ Signalling and Causes Scar Formation after Partial Hepatectomy
Source: PLoS One. 2014 May 6;9(5):e96576. doi: 10.1371/journal.pone.0096576 (PMC4011785; doi:10.1371/journal.pone.0096576)

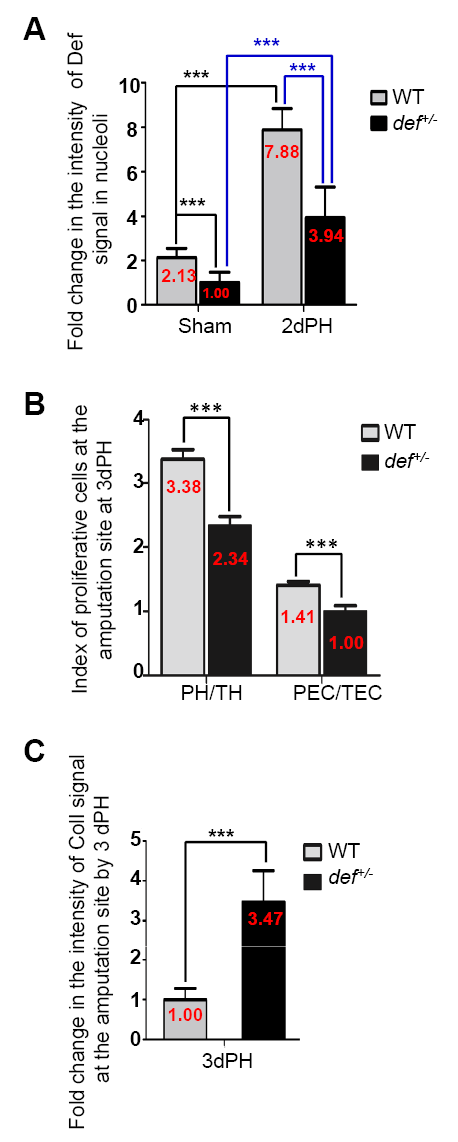

Supplement: Figure S1 — Statistic analysis of the signal intensity of the immunostaining for Def, PCNA and ColI, respectively. (A) Comparison of Def signal intensities among WT sham, def+/− sham, WT and def+/− at 2 dPH as showing in Figure 1C. The relative value of the Def signal intensity in def+/− was set at 1. (B) Comparison of the index of proliferating cells at the amputation site at 3 dPH as showing Figure 3C. Hepatocytes (Bhmt+ cells) and wound epidermal cells (Bhmt− cells) in the same areas at the amputation plane were counted. PCNA+ hepatocytes and wound epidermal cells were also counted. The average ratio of total PCNA+ hepatocytes (PH) to total hepatocytes (TH) and total PCNA+ epidermal cells (PEC) to total wound epidermal cells (TEC) in WT and def+/− were obtained, respectively. The value of PEC/TEC in def+/− was set as 1 for the convenience of comparison. Data were collected from three fish for each genotype in each case. (C) Comparison of the ColI signal intensities at the amputation site between WT and def+/− at 3dPH as showing in Figure 4B. The relative value of the ColI signal intensity in WT was set as 1. Ten sections from three WT or def+/− mutant fish were examined. In (A–C), the values plotted represent the means ± standard errors of the mean. The p-value was obtained by performing the two-tailed unpaired t-test. *** P<0.001; Student's t test. Signal intensity in each case was acquired by Photoshop based on the brightness of immunostaining of the targeted protein in a selected region. (TIF) [file pone.0096576.s001.tif]

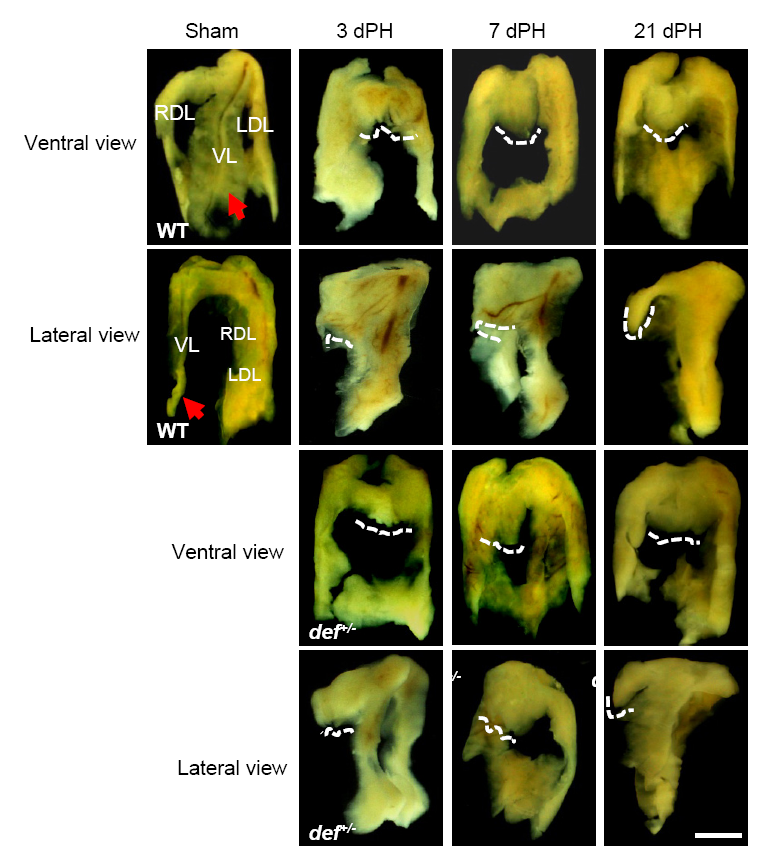

Supplement: Figure S2 — The def+/− mutant showed defective lobe structure recovery at the amputation site after PH. Lower magnifications of the ventral and lateral views of the gross morphology of the liver 3, 7 and 21 days after PH. The images for 7 days after PH correspond to the closer view of the amputation site shown in Figure 1d. The white, dashed line outlines the amputation site on the ventral tip. The red arrow highlights the ventral lobe in the sham control. VL: ventral lobe; LDL: left dorsal lobe; RDL: right dorsal lobe. Scale bar: 1 cm. (TIF) [file pone.0096576.s002.tif]

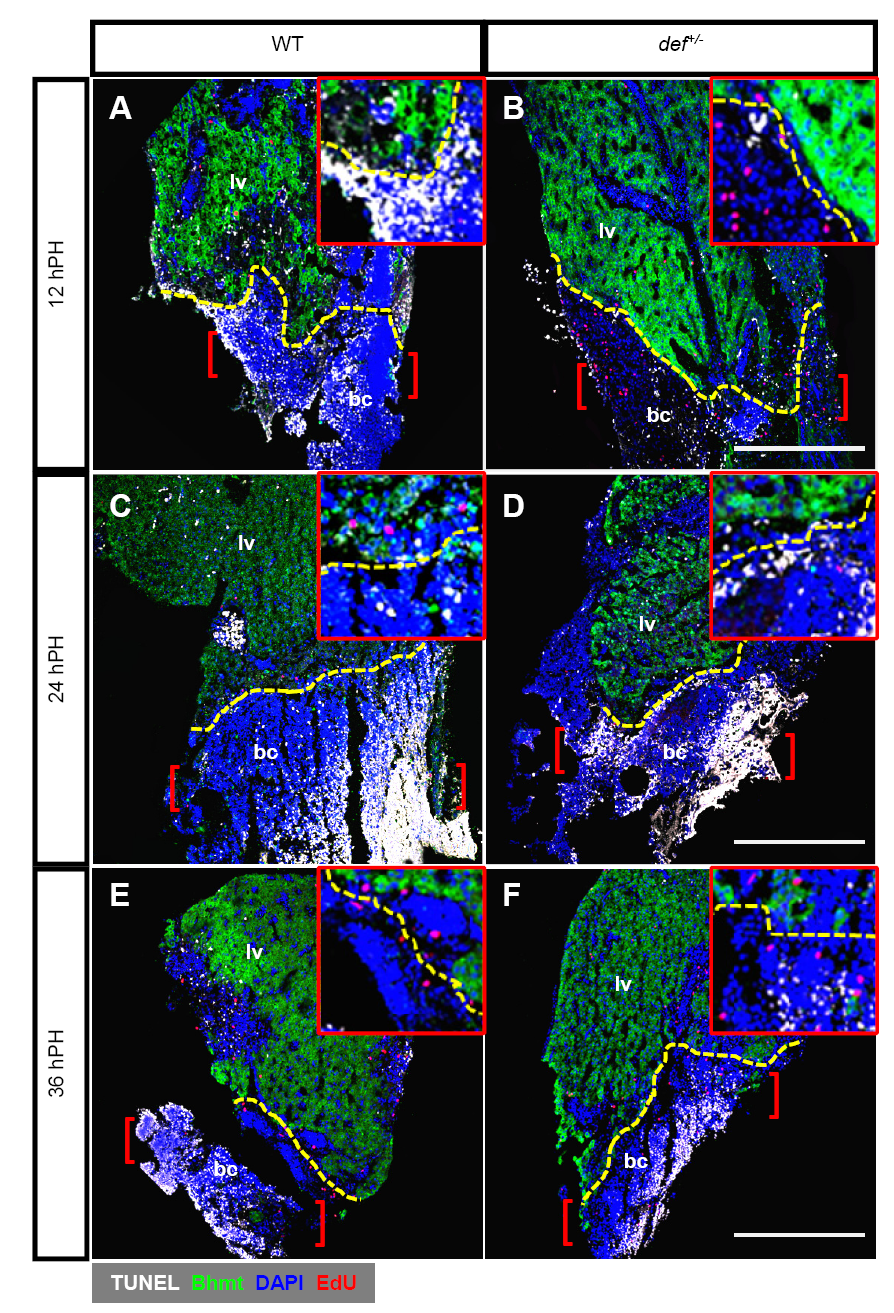

Supplement: Figure S3 — Comparison of the wound healing process between the wild-type and def+/− livers after PH. Frontal plane sections of the liver 12, 24 and 36 h after PH were stained for apoptotic cells (in white, using the TUNEL assay), proliferative cells (in red, by EdU incorporation) together with Bhmt (hepatocyte marker) (in green). DAPI was used to stain the nuclei (blue). All images show the part of the hepatic tissue adjacent to the amputation site. Yellow dashed lines outline the amputation site, while regions defined by red brackets represent the blood clot. (A–D) The blood clot was clearly formed in both the wild-type (A) and def+/− mutant (B) fish 12 h after PH. Cells in the blood clot underwent massive apoptosis 24 h after PH (C,D). (E,F) By 36 h after PH, the blood clot was detached from the liver mass in the wild-type fish (E), whereas it was still connected to the liver mass in the def+/− mutant (F). The inset, enlarged view corresponds to the amputation site (A–F). bc, blood clot; lv, liver tissue. Scale bar: 250 µm (A–F). (TIF) [file pone.0096576.s003.tif]

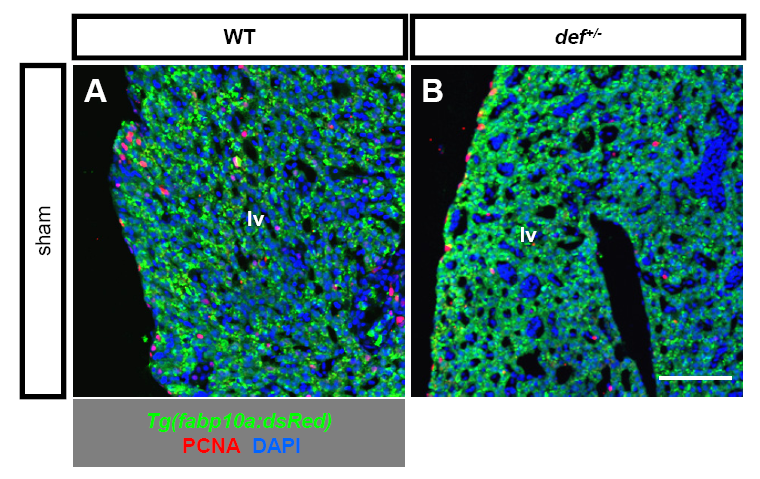

Supplement: Figure S4 — Comparison of cell proliferation in the adult liver in the wild-type and def+/− sham controls. Representative images of immunostaining of PCNA (in red) in the Tg(fabp10a:RFP) background, in which hepatocytes are genetically labelled by expressing the red fluorescent protein (in green), showed that proliferating cells were rarely detected along the epithelial edge of an adult liver in either the wild-type or def+/− mutant sham controls. Nuclei were stained with DAPI (blue). lv, liver tissue. Scale bar: 75 µm. (TIF) [file pone.0096576.s004.tif]

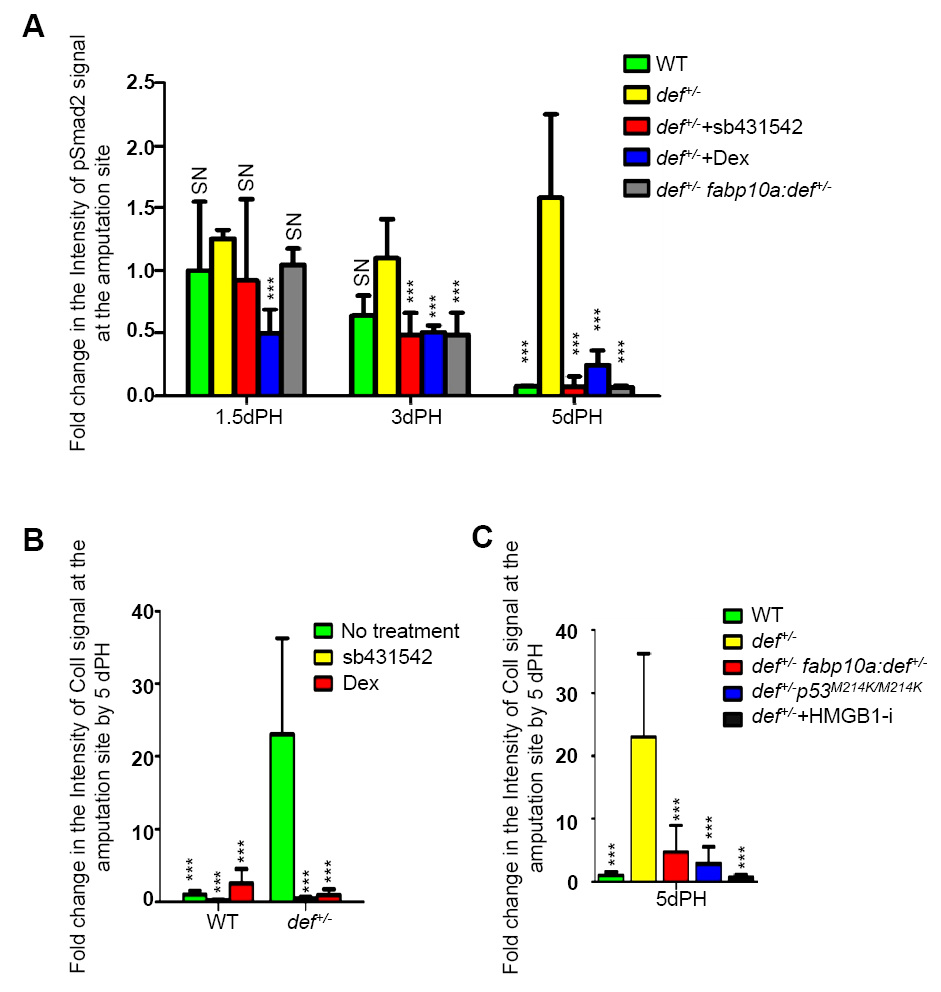

Supplement: Figure S5 — Statistic analysis of the effect of different treatment on the signal intensity of the immunostaining for pSmad2 and ColI at the amputation site, respectively. (A). Comparison of the pSmad2 signal intensities in WT, def+/−, def+/− treated with SB431542 or Dex, and def+/− Tg(fabp10a:def) at 1.5, 3 and 5dPH, respectively, as showing in Figure 6A–D and Figure 10A. Ten sections from three fish for each genotype in each case were examined. (B) Comparison of the ColI signal intensities in WT, def+/−, WT or def+/− treated with SB431542 or Dex at 5dPH as showing in Figure 6E and 6F. Ten sections from three fish in each case were examined. (C) Comparison of the ColI signal intensities in WT, def+/−, def+/− Tg(fabp10a:def), def+/−p53M214K/M214K or def+/− treated with HMGB1-i at 5dPH as showing in Figure 10B and 10E and Figure 11B. Ten sections from three fish for each genotype in each case were examined. In (A–C), the values plotted represent the means ± standard errors of the mean. The p-value was obtained by performing the two-tailed unpaired t-test. *** P<0.001; NS: non significant. Signal intensity in each case was acquired by Photoshop based on the brightness of immunostaining of the targeted protein in a selected region. (TIF) [file pone.0096576.s005.tif]

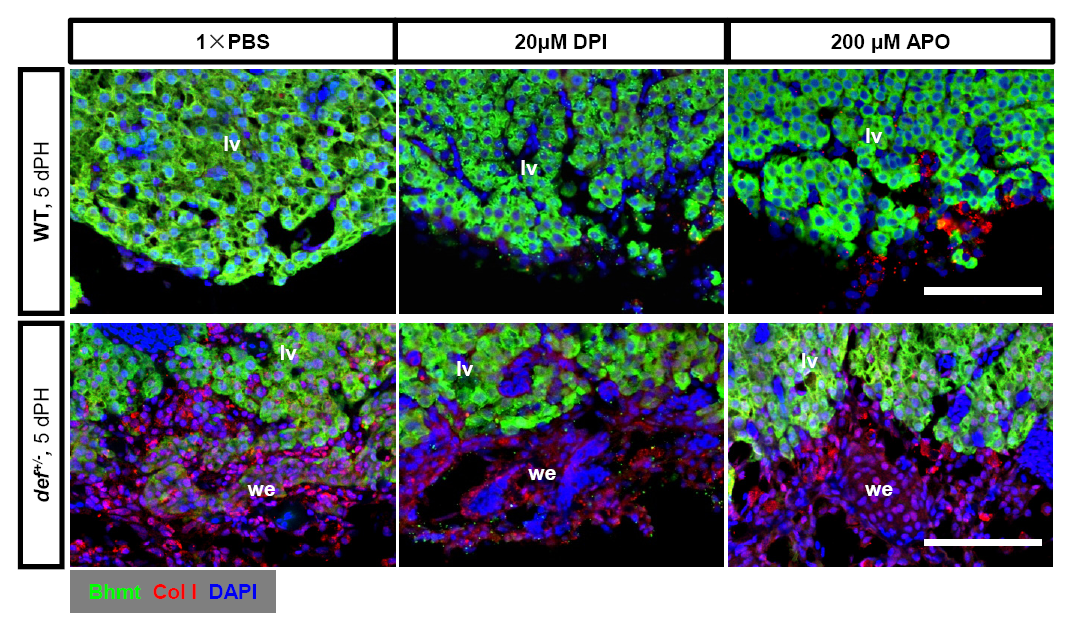

Supplement: Figure S6 — ColI deposition at the amputation site in def+/− is not mediated by the reactive oxygen species signalling pathway. Representative images of co-immunostaining of ColI (red) and Bhmt (green) in the wound epidermis in wild-type, def+/− and def+/− fish treated with diphenyleneiodonium (DPI) or apocynin (APO), two inhibitors of the reactive oxygen species signalling, 5 days after PH. Nuclei were stained with DAPI (blue). lv, liver tissue; we, wound epidermis. Scale bar: 75 µm. (TIF) [file pone.0096576.s006.tif]

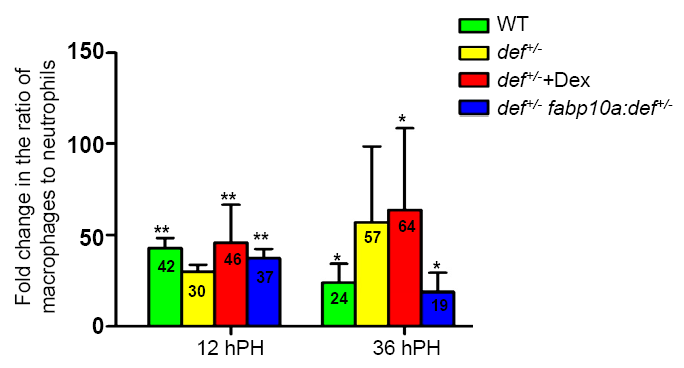

Supplement: Figure S7 — Comparison of the ratio of macrophages to neutrophils at the amputation site. The number of macrophages and neutrophils at the amputation site were counted, respectively, in WT, def+/−, def+/− treated with Dex, and in def+/− Tg(fabp10a:def) at 12 and 36 hPH as showing Figure 8. Ten sections from three fish in each case were examined and the ratio of macrophages to neutrophils was obtained for each section to minimize the variation caused by sectioning. The values plotted represent the means ± standard errors of the mean. The p-value was obtained by performing the two-tailed unpaired t-test. * P<0.05,** P<0.01. Signal intensity in each case was acquired by Photoshop based on the brightness of immunostaining of the targeted protein in a selected region. (TIF) [file pone.0096576.s007.tif]
